# Mapping livestock density distribution in the Selenge River Basin of Mongolia using random forest

**DOI:** 10.1038/s41598-024-61959-7

**Published:** 2024-05-15

**Authors:** Yaping Liu, Juanle Wang, Keming Yang, Altansukh Ochir

**Affiliations:** 1 College of Geoscience and Surveying Engineering, China University of Mining & Technology (Beijing), Beijing, 100083 China; 2 State Key Laboratory of Resources and Environmental Information System, Institute of Geographic Sciences and Natural Resources Research, Chinese Academy of Sciences, Beijing, 100101 China; 3 College of Resources and Environment, University of Chinese Academy of Sciences, Beijing, 100049 China; 4 Jiangsu Center for Collaborative Innovation in Geographical Information Resource Development and Application, Nanjing, 210023 China; 5 Environmental Engineering Laboratory, Department of Environment and Forest Engineering, School of Engineering and Applied Sciences and Institute for Sustainable Development, National University of Mongolia, Ulaanbaatar, 14201 Mongolia

**Keywords:** Grassland ecology, Environmental sciences

## Abstract

Mapping dynamically distributed livestock in the vast steppe area based on statistical data collected by administrative units is very difficult as it is limited by the quality of statistical data and local geographical environment factors. While, spatial mapping of livestock gridded data is critical and necessary for animal husbandry management, which can be easily integrated and analyzed with other natural environment data. Facing this challenge, this study introduces a spatialization method using random forest (RF) in the Selenge River Basin, which is the main animal husbandry region in Mongolia. A spatialized model was constructed based on the RF to obtain high-resolution gridded distribution data of total livestock, sheep & goats, cattle, and horses. The contribution of factors influencing the spatial distribution of livestock was quantitatively analyzed. The predicted results showed that (1) it has high livestock densities in the southwestern regions and low in the northern regions of the Selenge River Basin; (2) the sheep & goats density was mainly concentrated in 0–125 sheep/km^2^, and the high-density area was mainly distributed in Khuvsgul, Arkhangai, Bulgan and part soums of Orkhon; (3) horses and cattle density were concentrated in 0–25 head/km^2^, mainly distributed in the southwest and central parts of the basin, with few high-density areas. This indicates that the RF simulation results effectively depict the characteristics of Selenge River Basin. Further study supported by Geodetector showed human activity was the main driver of livestock distribution in the basin. This study is expected to provide fundamental support for the precise regulation of animal husbandry in the Mongolian Plateau or other large steppe regions worldwide.

## Introduction

Livestock serves as a direct carrier of human disturbance to the ecosystem and is a key factor in the stabilization of grassland ecosystems^1–3^, with important implications for global food security and environmental sustainability^4^. In recent decades, animal husbandry has developed rapidly in many countries due to government policy changes. However, significant changes in livestock numbers and structure have introduced considerable uncertainty in assessments of the environmental impact and feedback associated with analyzing animal husbandry systems to a large extent, particularly regarding the nitrogen and phosphorus cycles, greenhouse gas emissions and ecosystem destruction^5,6^. Grassland degradation owing to overgrazing is a global issue that directly affects the healthy development of grasslands^7,8^. The spatial distribution of livestock can reflect forage utilization and grazing pressures. In contrast to other factors affecting grassland ecosystem protection, livestock distribution is considered to be the only controllable and predictable factor^9^. Therefore, accurately determining the spatial distribution of livestock is essential for improving scientific understanding and informing practical applications for the management of grassland ecosystems. However, the geographical spatial heterogeneity of livestock distribution cannot be expressed in detail because livestock is evenly distributed within the same administrative unit in traditional statistical style.

In response, methods to simulate the spatial distribution of livestock in a certain time and space using parameters and models have emerged. Livestock density and geospatial distribution patterns can be reflected in detail by spatially discretizing livestock statistics. Remote sensing technology provides an important means for monitoring the spatial distribution of livestock at different scales. Multi-layer regression models and machine learning algorithms have been used to realize the transformation of livestock statistics from administrative units into grid-scale, which is achieved by establishing the relationship between livestock statistics and environmental factors to enable the geographical representation of livestock distribution information. Selection of environmental factors is vital in simulating the spatial distribution of livestock. Climatic and environmental conditions, livestock foraging behavior, and herders’ behavioral habits can contribute to revealing the spatial heterogeneity of livestock distribution^10–12^. For example, large concentrations of livestock are observed in nearby areas rich in water and pasture resources, and plains or low-lying areas are preferred habitats for livestock^13^. Furthermore, the livestock distribution is associated with herders’ activities, as most livestock moves in proximity to settlements^14,15^. How to use the spatial model to integrate the spatial heterogeneity of various environmental factors and reasonably simulate the spatial distribution of all kinds of livestock is an important issue.

The Food and Agriculture Organization of the United Nations (FAO) first launched the Gridded Livestock of the World (GLW) project. The global livestock density distribution dataset for 2002 (GLW1) and the global distribution map for cattle, pigs, and chickens for 2006 (GLW2) were developed based on multi-layer regression models to link livestock density with predictors^16,17^. During this period, Prosser constructed a model that related poultry census data with agroecological variables and presented the first high-resolution spatial density-gridded map of poultry in China^18^. In recent years, advanced geographic computing technologies, including using Random forest (RF), have emerged to solve the problems associated withthe geographical representation of livestock. Gilbert used RF algorithms in combination with predictors to obtain global-scale 10-km livestock distribution data (GLW3)^19^, demonstrating improved the simulation accuracy. Li et al. established the relationship between various environmental factors (such as land, terrain, climate, and socioeconomic factors) and livestock density using the RF, and constructed a spatial distribution map of cattle and sheep in western China at a 1 km resolution^20^. Some scholars have realized the acquisition of livestock density gridded datasets in Qinghai-Tibet Plateau and Kazakhstan based on the RF, which can bridge the gap in the dynamic monitoring of the spatial distribution of livestock density in the region over a long period^21,22^. Cheng et al. used RF to downgrade reared pigs from the administrative level to 30 × 30 arcsec (about 131 km) resolution^23^. Compared to the traditional multiple regression model, the RF has a faster training speed, can process large-scale and complex geographic data, and can estimate the relative importance of each feature^19,24^. Currently, this model is utilized in simulation research on the spatial distribution of population, crops, livestock, and soil organic matter^25,26^.

The Selenge River Basin is an important freshwater source for Lake Baikal and is crucial to global ecological security. Abundant water and forage resources have facilitated the rapid development of animal husbandry in the region. However, the ecological dynamics of the basin are complex and sensitive to global change. Approximately 90% of the land in this region at risk of desertification due to overgrazing^27^, which has become a major challenge for the sustainable development of grassland. Therefore, a comprehensive understanding and investigation of livestock distribution patterns in the Selenge River Basin is essential for the scientific management of animal husbandry resources, the evaluation of grassland health, and the formulation of sustainable development strategies. However, studies on the spatial distribution of livestock in the Selenge River Basin of Mongolia are relatively limited. Although some studies have focused on changes in livestock numbers and specific distribution characteristics, the spatial heterogeneity of livestock and its influencing factors are not well understood. Thus, it is necessary to strengthen the study of livestock spatial simulation in the Selenge River Basin and further understand the factors affecting the spatial distribution of livestock.

Therefore, this study compiled statistical data on livestock at the soum scale in the Selenge River Basin to reveal the geographical distribution pattern of livestock density in this region. The main objectives of the study were to: (1) decompose the soum-level livestock density to the grid scale using the RF model, obtain high-precision gridded distribution data for total livestock, sheep & goats, cattle, and horses, and fill in the gaps of the livestock densities gridded data in 2020; (2) identify the drivers of livestock distribution and quantifying their contributions; (3) provide a reference method for mapping the spatial distribution of livestock density not only in Mongolian Plateau but also in other steppe areas.

## Results

### Livestock spatialization results

**Figure 1:**
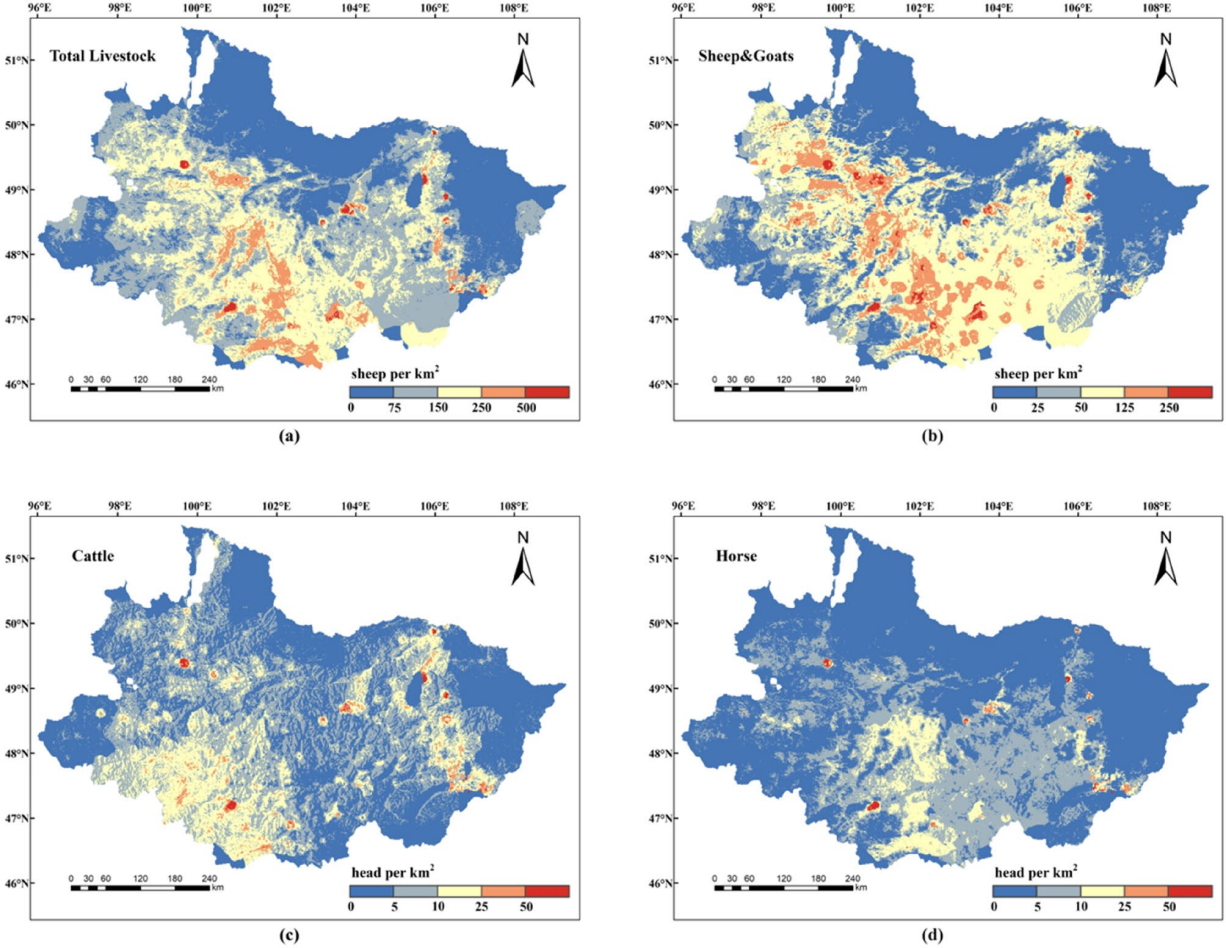
Simulation of the spatial distribution of livestock in the Selenge River Basin: (**a**) total livestock, (**b**) sheep & goats, (**c**) cattle, and (**d**) horse. The maps were created in ESRI ArcMap 10.2 (https://support.esri.com/zh-cn/products/desktop/arcgis-desktop/arcmap/10-2-2).

After repeated training, the accuracy of the model after determining the optimal parameters was as follows: 92.88% for total livestock, 91.35% for sheep & goats, 89.96% for cattle, and 89.86% for horses. The trained model was applied to the grid scale for predicting the density values of livestock. Figure 1 shows the distribution of various livestock gridded densities in the Selenge River Basin in Mongolia.

The simulation of livestock spatial distribution based on the RF model finely depicts the distribution characteristics of high livestock density in the southwest and low livestock density in the north of the Selenge River Basin of Mongolia in 2020. Livestock in this area showed a concentrated distribution trend, and areas of high livestock density showed a continuous sheet-shaped distribution. The spatial distribution trend of total livestock was roughly similar to that of sheep & goats, which was due to the merging of sheep and goats in this study to simulate the combined spatial distribution of sheep & goats in the basin. The number of sheep & goats in this region accounted for 49% of the total livestock, approximately half of the total livestock, which was well reproduced in the simulation map.

**Figure 2:**
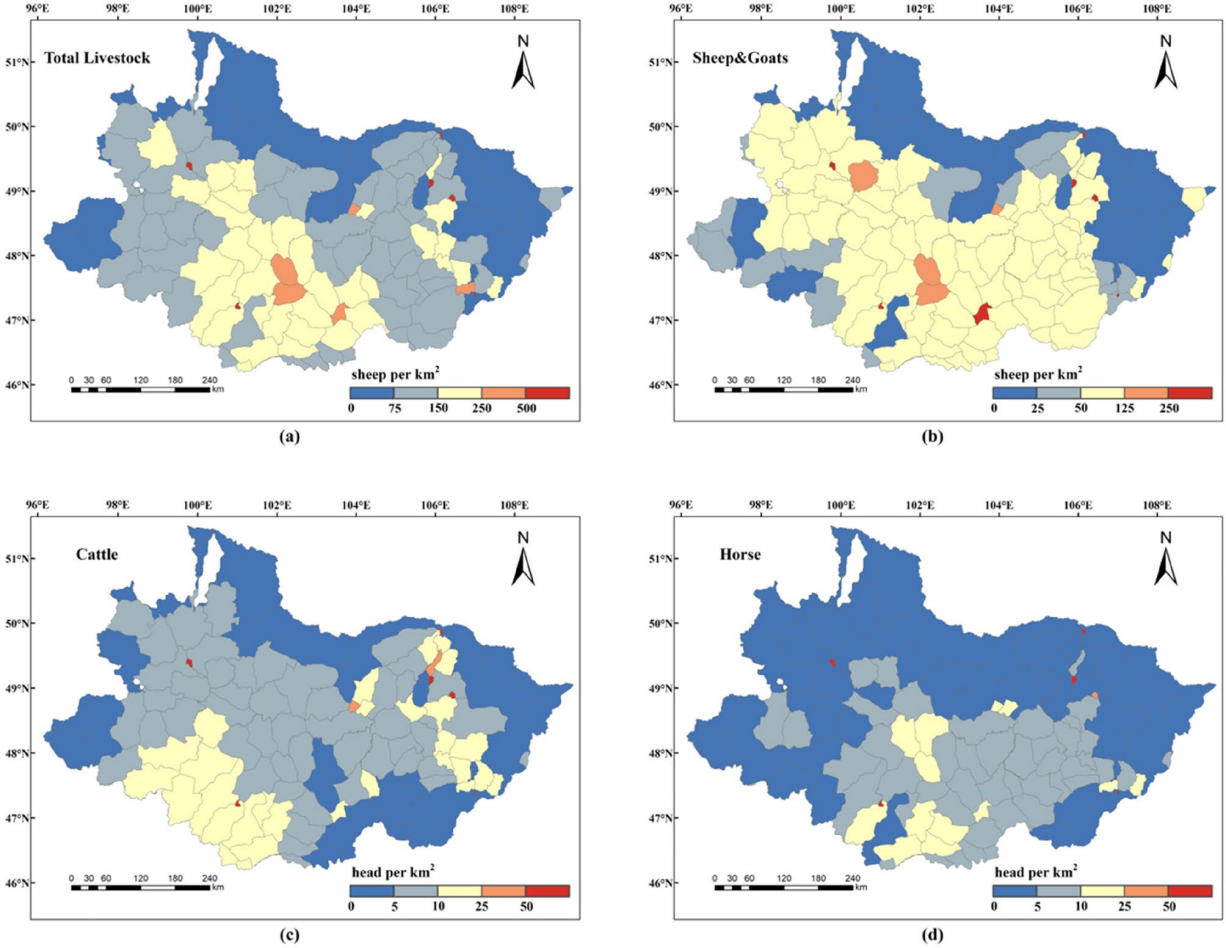
Spatial distribution of livestock statistics of each soum in the Selenge River Basin: (**a**) total livestock, (**b**) sheep & goats, (**c**) cattle, and (**d**) horse. The maps were created in ESRI ArcMap 10.2 (https://support.esri.com/zh-cn/products/desktop/arcgis-desktop/arcmap/10-2-2).

To make a more obvious comparison, Fig. 2 depicts the spatial distribution map of the total livestock, sheep & goats, cattle, and horse densities of statistical data in each soum. As shown in the figure the spatial distribution map of various livestock densities in the Selenge River Basin simulated in this study was consistent with the general distribution trend of statistical data. Among them, the total livestock density was concentrated in 0–250 sheep/km^2^, and the sheep & goats density was mainly concentrated in 0–125 sheep/km^2^. The number of horses and cattle in the basin was relatively small, and the density of horses and cattle was mainly concentrated in 0–25 head/km^2^. In terms of spatial distribution, sheep & goats were mainly concentrated in southern Khuvsgul, northeastern Arkhangai, the southern Bulgan, the Tuv, the western Darkhan-Uul, and the northern Uvurkhangai. High density of sheep & goats was found in Murun, Tosontsengel, Alag-Erdene, and Ikh-Uul in Khuvsgul, and especially in Ulziit and Ugiinuur in eastern Arkhangai, Rashaant in Bulgan and Orkhon. These areas have relatively flat terrain and abundant water resources, making them suitable for agricultural production. However, the density of sheep & goats was relatively low in the northern and eastern regions of the basin. Areas with a high density of cattle included Arkhangai in the southwestern basin and Ulaanbaatar and to a lesser extent in the north. The number of horses was significantly lower than that of cattle and sheep & goats, which is consistent with the statistical data. The density of horses was relatively high in Saikhan of Bulgan, Khairkhan in northern Arkhangai, and Khujirt and Bat-Ulzii in Uvurkhangai, with lower densities in other areas.

In general, regions with high livestock density were mainly concentrated in Arkhangai and Uvurkhangai, while Orkhon and Darkhan are tourist cities and agricultural provinces respectively. Arkhangai is located in the Khangai Mountain range, has strong water conservation ability, and is the birthplace of the Selenge River tributaries Orkhon and Hanuy River, with suitable climatic conditions, which provides a favorable habitat for livestock to survive.

### Importance analysis of environmental factors

**Figure 3:**
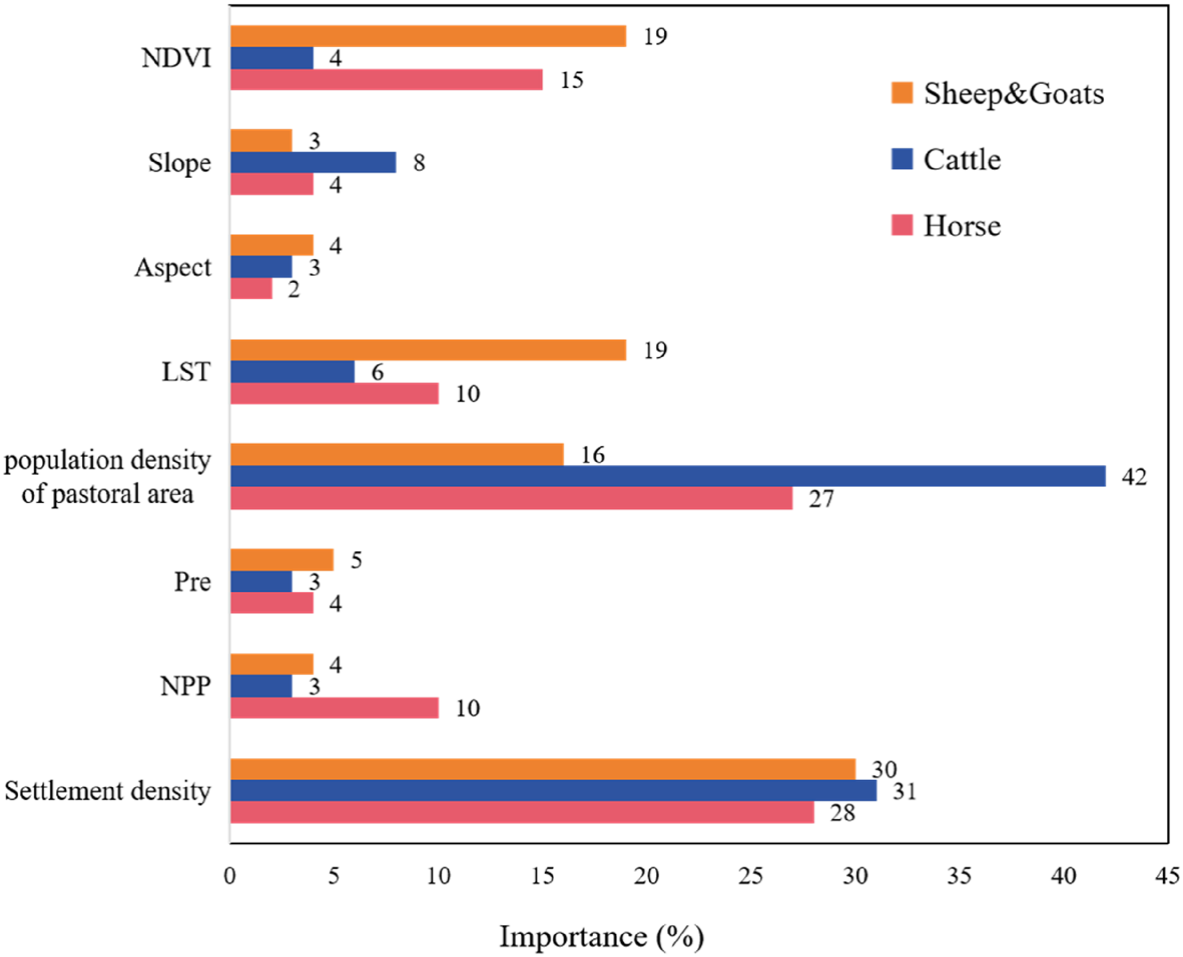
Evaluation of the importance of environmental factors in RF.

The environmental variables for sheep & goats, cattle, and horses were conducted individually because the total livestock density data used in the simulation of this study was the sum of all types of livestock units converted in the Selenge River Basin, which is comprehensive. OOB data was used to construct the RF model for feature importance analysis. Figure 3 shows the extent of the influence of variable factors on livestock density in the RF model. The higher the value, the greater the contribution of the variable to the RF regression, and the better it can explain the dependent variable. The population density of pastoral area, settlement density and NDVI were the factors with high contributions to the model.

The factor detector yielded the degree of explanation q of each environmental factor on the simulated density of sheep & goats, cattle and horses (Table 1). The selected detector factors affecting the distribution of simulated livestock densities in the Selenge River Basin were statistically significant (p < 0.05) for all q-statistics values except slope and aspect.Among them, the population density of pasture area and settlement density had the strongest explanatory power on the spatial heterogeneity of the densities of sheep & goats, horses, and cattle, indicating that the population was an important influence on the distribution of livestock, especially on cattle. NDVI and NPP also had strong explanatory power on the distribution of each type of livestock, and especially had a greater influence on the spatial distribution of sheep & goats. Precipitation and land surface temperature had some driving effect on the density of each type of livestock, but the explanatory power was small. The weakest explanatory power for densities of sheep & goats and horse was aspect, with q-values of 0.08 and 0.05, and the weakest explanatory power for cattle densities was land surface temperature, with q-value of 0.05. Slope direction was the weakest explanatory power among all the drivers, and the high and low densities of livestock in the Selenge River Basin were relatively weakly influenced by the topography.

**Table 1:** The q-statistics values of the factors.

	NDVI	Slope	Aspect	LST	Population density of pastoral area	Pre	NPP	Settlement density
Sheep & goats	0.26	0.18	0.08	0.23	0.33	0.19	0.24	0.33
Cattle	0.18	0.07	0.06	0.05	0.53	0.15	0.21	0.53
Horse	0.19	0.17	0.05	0.15	0.47	0.16	0.23	0.49

**Figure 4:**
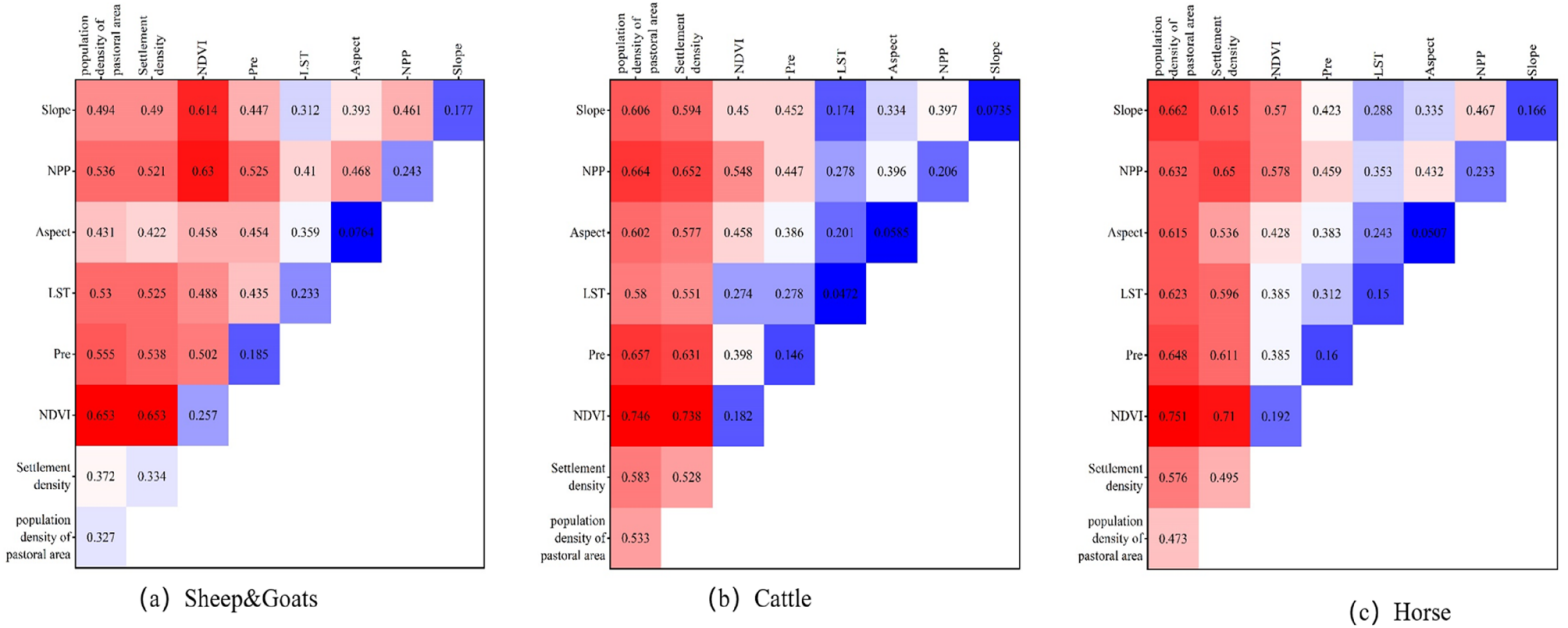
Plot of the results of the interaction between two variables affecting livestock density.

We used the interaction detector to explore the effects of the interaction of environmental factors on the density of various types of livestock (Fig. 4). The results showed that the effects of each environmental factor on livestock density were not independent, and any two factors interacting with each other contributed more to livestock density than a single factor. This indicates that the explanatory power of various types of livestock densities was further enhanced by the interaction of factors compared with that of a single factor. Among them, the explanatory power of the interaction of NDVI with the population density of pasture area and settlement density on sheep & goats, cattle and horse densities exceeded 0.6 and was greater than that of the interaction between other factors. The interactions of all other factors with pastoral population density and settlement density were significantly larger than their q-values when they were independent factors, supporting that population was the main factor influencing livestock distribution in the region. The interaction between NDVI and pasture population density was the main factor with the strongest explanatory power for sheep, horse, and cattle densities, with q-values of 0.653, 0.746, and 0.751, respectively. The interaction between slope and aspect and the factors also enhanced their explanatory power for livestock density.

### Comparative analysis of accuracy verification

To verify the accuracy of the simulation results in this study, the simulated livestock density in each soum was counted in zones and compared with the statistical data for verification. To ensure the accuracy and authenticity of the results, the data of zero livestock and incomplete administrative divisions in the statistical data were excluded, and 99 soum-level data samples were retained. We calculated the RMSE, MAE, and R^2^ among the four livestock density datasets obtained in this study and the statistical data, where the units of MAE and RMSE are sheep units/km^2^. R^2^ indicates the extent of closeness between the predicted values and statistical values, and the closer R^2^ is to 1, the more accurate the predicted result. The calculated values of the accuracy verification metrics are shown in Table 2.

**Table 2:** Calculation results of accuracy validation metrics.

Datasets	MAE	RMSE	R^2^
Total livestock	33.3847	224.7337	0.9588
Sheep & goats	13.5597	86.1839	0.9713
Cattle	2.6146	18.0546	0.9184
Horse	1.6426	10.8959	0.9690

**Figure 5:**
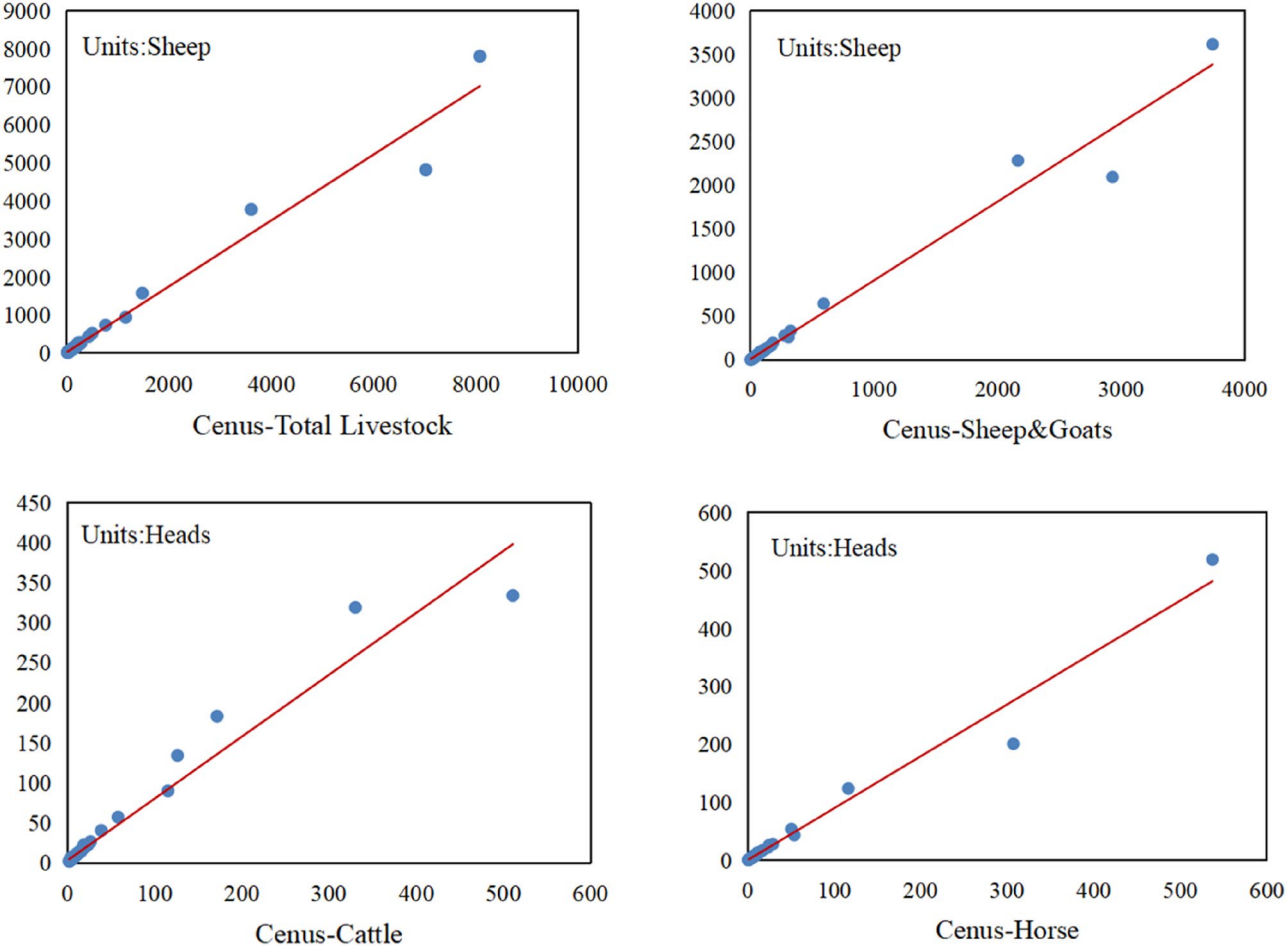
Validation of the grazing spatialization dataset at the soum level.

The grid-scale simulation results showed that the livestock density values simulated by RF had good consistency with the livestock density statistics of the soum level. Except for a few soum that exhibited overestimation or underestimation, the overall performance was better. The R^2^ of sheep & goats was the highest at 0.9713, while the R^2^ of cattle was lower, but the overall R^2^ was above 0.9, indicating that the linear fitting result was good, as shown in Fig. 5.

**Figure 6:**
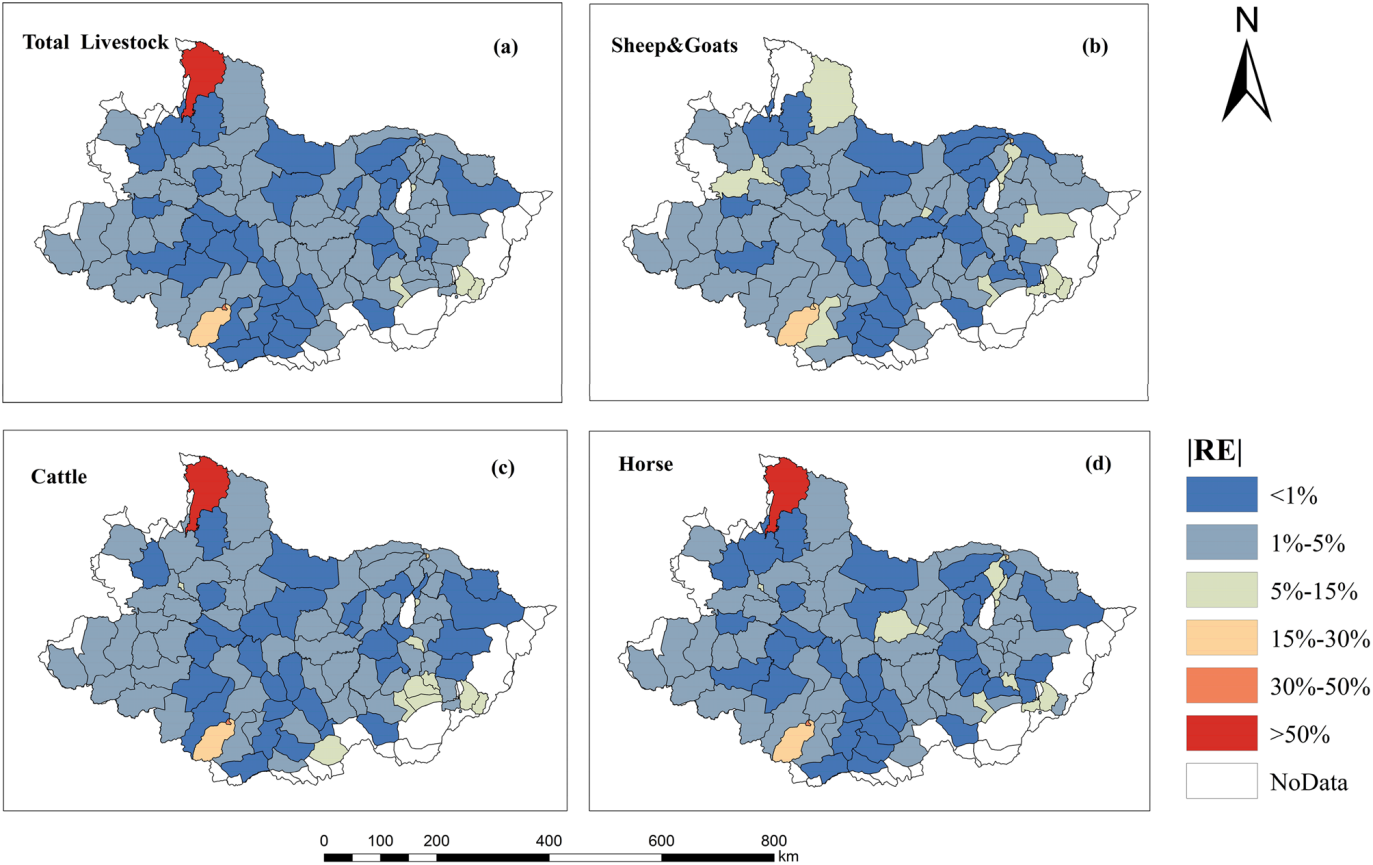
Relative error geospatial distribution map. The maps were created in ESRI ArcMap 10.2 (https://support.esri.com/zh-cn/products/desktop/arcgis-desktop/arcmap/10-2-2).

To intuitively understand the geographical distribution of the relative errors, we took the four livestock spatialization results obtained in this study as an example, and drew the distribution map of the relative errors at the soum scale in the Selenge River Basin (Fig. 6). The relative error of most soums in the whole basin was < 5% and the simulated value was similar to the statistical data. Moreover, the spatial simulation results for the four livestock types showed similar error distributions, and the simulated results for the entire basin were within a relatively reasonable range. Part of Ulaan-Uul in Kusugul province was delineated as an unsuitable area for livestock when the spatial distribution of livestock was modeled, resulting in a large error of > 50%. Moreover, the error of the four simulation results for Tsenkher and Tsetserleg in Arkhangai was between 15 and 30%, exhibiting overestimated livestock density. Overall, the simulation results of the spatial distribution of livestock in this study were consistent with the actual situation.

## Discussion

### RF adaptation study

Past research on the Selenge River Basin of Mongolia predominantly focused on ecological environmental assessment and geographic information extraction, while most livestock-related research currently focuses on infectious disease assessment and econometrics, with only a few studies attempting to shed light on the spatial distribution of livestock in this region. In this study, we focused on revealing the spatial distribution pattern of livestock density in the basin in 2020 to address the information gap regarding the geographical spatial distribution of livestock in the basin to determine the requirements for ecological development in this basin.

The livestock spatial framework based on RF can further improve the simulation accuracy. Compared to previous studies in the field^17,19^, the spatial model we used showed a higher classification accuracy and was better able to analyze the complex interrelationships between variables^28–30^. Moreover, it can flexibly analyze different types of statistical data, including regression, classification, and unsupervised learning, and analyze the significance of the factor variables as needed. To confirm the accuracy of the simulated data, the livestock density gridded data created based on the RF model were compared and examined using the statistical data. The results showed that, while some soums exhibited slight overestimations of livestock density, the relative error for the majority of soums across the entire basin was < 5%, the simulated value was comparable to the statistical value, and the spatial distribution of the acquired livestock was consistent with the statistical data. Overall, the RF model was found to be capable of accurately identifying the geographic distribution of livestock.

### Characterization of the spatial distribution of livestock

The simulation results for livestock distribution in this study are consistent with the trend reported by Saizen^9^ and roughly depict the areas where sheep & goats, cattle, and horses congregate in the basin, highlighting the areas with favorable conditions for livestock survival. The principal livestock of the Selenge River Basin including sheep & goats, cattle, and horses, exhibit varying distributions across certain provinces, showing a spatial clustering state, demonstrably representing genuine spatial dependence patterns inherent in livestock distribution^21^. They were mainly distributed in Khuvsgul, eastern Darkhan, and the middle-lower parts of the Khangai region upstream of the basin. A common feature of these regions is the presence of numerous rivers and lakes, and the predominant grassland type is typical grassland, which provides livestock with forage and other feed. Under the influence of Mongolia’s economic and population management systems, livestock has become particularly abundant in Uvurkhangai and Arkhangai. Khangai and the central areas witnessed a major influx of herders in the middle of the 1990s, which aided the rapid growth of the livestock sector^31^. In 2020, 41.62% of the population of Khangai was engaged in animal husbandry, which was the highest in Mongolia^32^. This reflects that livestock were mainly concentrated in areas marked by human activity, which are related to traditional grazing methods in Mongolia. The Darkhan region has large numbers of livestock with sufficient conditions for intensive animal husbandry. Regional variations in livestock spatial distribution are attributable to their nutritional preferences, production adaptability, and human activities^20^. Sheep & goats, which can adapt to various weather conditions and have excellent environmental adaptability, were more densely concentrated in the middle and lower reaches of the basin. Cattle were mainly distributed in the southwestern area of the basin, and horses show high-density concentrations mainly in the eastern region, where cattle are similarly dense, with a small distribution area and low livestock density.

### Analysis of factors influencing the spatial distribution of livestock

Based on the importance of each environmental factor output by RF, we preliminarily determined that the population density of the pastoral area, settlement density, and NDVI contributed more the most to the model (Fig. 3). To further quantify the effects of various environmental factors on the density of livestock, we used the factor detector and the interaction detector to detect the explanatory power of various environmental factors on the density of sheep & goats, cattle and horses. Compared with a single factor, the explanatory power of each type of livestock density was further enhanced under the interaction of factors. The population density of pastoral areas and settlement density were the strongest drivers of all types of livestock densities, whether it is a single factor or the interaction with other environmental factors. In Mongolia, grazing continues to be performed in a traditional family-based manner as an aspect of nomadic animal husbandry in grasslands. One-third of the region's population maintains a pastoral lifestyle^33^, and the human driving force is large. The distribution and living conditions of livestock are affected by human activities, such as grazing techniques, herdsmen density, and animal husbandry management. In pastoral areas, in addition to social factors such as population density of pastoral areas and settlement density, livestock activities are constrained by natural environmental factors such as land surface temperature, precipitation, and terrain. Suitable temperature and climate conditions help the growth of pasture and provide excellent environmental conditions for livestock survival activities, and NDVI and NPP can provide pasture resources for livestock activities. Livestock distribution is influenced by both natural and socio-economic factors^34^. In general, social variables exerted a greater influence on the distribution of livestock than other natural factors, consistent with the development of animal husbandry in Mongolia. Simultaneously, each variable had a different effect on the distribution of different livestock species, indicating that the distribution of different livestock species is affected by different factors. The factors selected in this study are representative of the many factors that influence livestock distribution. Therefore, a reasonable introduction of environmental factors that are closely associated with livestock distribution is conducive to improving the accuracy of livestock spatial simulation.

### Relevant recommendations

Overgrazing is the main cause of grassland degradation in the Selenge River Basin^35^; however, traditional livestock data cannot accurately reveal the geographical distribution pattern of livestock or determine the extent of overloaded grazing. In 2020, the number of livestock in Mongolia reached approximately 67.1 million, representing a decrease from the previous year due to the effects of epidemics, natural disasters, and overgrazing of grasslands. The spatial distribution maps of the various livestock obtained in this study hold certain policy significance for the development and management of animal husbandry in the Selenge River Basin. Areas where livestock mainly gather such as the Khangai region and Khuvsgul regions, are experiencing severe land degradation due to overgrazing, which has overloaded grasslands and hindered their ability to provide sufficient forage for livestock survival, which in turn has affected the growth of the livestock industry. Therefore, further recommendations for optimizing the livestock sector were made based on the spatial distribution of livestock:

1. Optimizing the herd structure. Some studies have shown that changes in the proportion of livestock within a structure can substantially influence the extent of desertification^27^. Considering the large number of sheep in this basin, it is possible to promote the production of sheep products in accordance with the market demand, thereby lowering the number of sheep on hand and minimizing pasture damage. The number of cattle and horses can be increased to increase the efficient utilization of pastures while maintaining a reasonable ecological carrying capacity.
2. Maintaining mobile grazing operations. Mobile grazing can effectively alleviate grassland degradation^36^. Livestock activity on different ranges of grasslands can not only effectively reduce trampling back and forth on the grassland, but also alleviate grassland degradation and damage to the surrounding water environment.
3. Implementing measures to protect the grassland ecological environment. Scientific grassland management techniques, such as rotational grazing, grassland restoration, and ecological protection measures, can be used to prevent overgrazing and the deterioration of the grasslands, as well as to maintain the capacity of the grassland for sustainable use.

### Limitations and future work

This study provided a feasible method for livestock dynamic mapping by gridded pixels in Mongolia. However, there were some limitations in this study. The livestock statistics at the sub-soum level are not all publicly available, and administrative division vector data are missing, thus limiting the analysis to input data at the soum scale and preventing data validation at the sub-soum. Since the statistical values of each environmental factor at the grid scale are different from the characteristic statistical values at the soum scale, when applying the soum-scale training model to the grid scale will lead to prediction errors in cross-scale modeling. Although we utilized soum statistics for redistribution, the scale mismatch was not fundamentally addressed. In future studies, we will explore solutions for the scale mismatch problem in the downscaling process, to reduce the scale difference between administrative unit-scale and grid-scale feature representation. And we will conduct a long-term series study next step to cover the past 30 years changes of livestock in this region, and further distinguish goats and sheep. In addition, we will try to select more abundant geographical factors, such as the percentage of cropland and grassland, to further optimize the model.

## Conclusions

Livestock spatial distribution simulation is essential for the generation of livestock gridded data with spatial geographical significance through the use of gridded technology, which can serve as a foundation for spatial management and decision support in animal husbandry, optimize resource utilization, and provide scientific data for the sustainable development of grasslands. In this study, we established a statistical correlation between livestock inventory data and geographic information, such as land use, population, terrain, and climate based on RF in 2020, and precisely described the spatial distribution characteristics of livestock in the Selenge River Basin. The most notable characteristic of the livestock density distribution in the basin was a high density in the southwest and a low density in the north. In terms of herd structure, sheep & goats were the most abundant livestock type in the basin, with relatively small numbers of horses and cattle. Areas with sheep & goats density exceeding 50 sheep/km^2^ are concentrated in the middle of the basin, mainly including Khuvsgul, Arkhangai, Bulgan, and part soums of Orkhon, accounting for over half of the entire basin. The distribution density of horses and cattle was mainly concentrated in 0–25 head/km^2^ and was mainly distributed in the southwest and central parts of the basin respectively. Simultaneously, the population density of pastoral areas in the basin is the most important factor in explaining the spatial distribution of livestock in the basin, which also reflects the traditional grazing practices in Mongolia to a certain extent.

## Methods

### Study area

**Figure 7:**
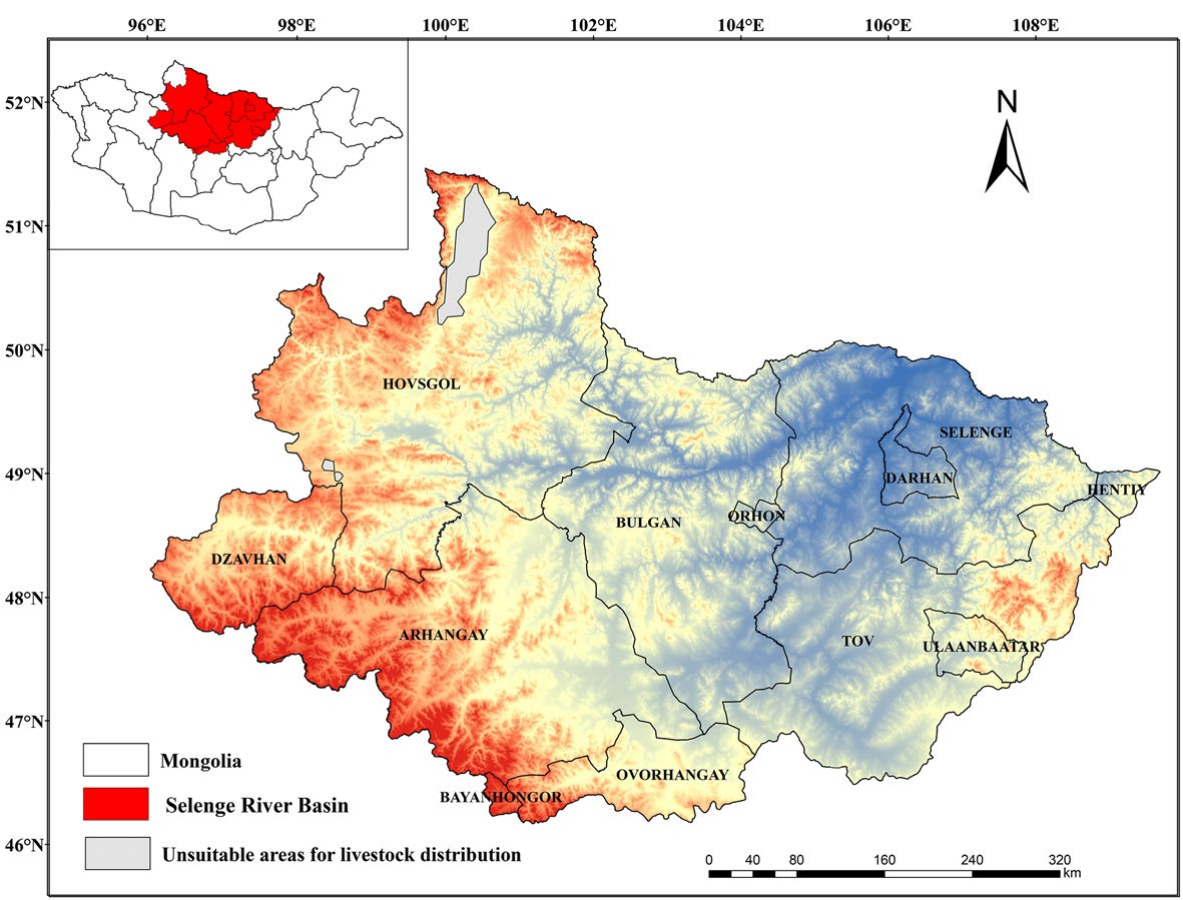
Geographic location of the study area. The map was created in ESRI ArcMap 10.2 (https://support.esri.com/zh-cn/products/desktop/arcgis-desktop/arcmap/10-2-2).

The Selenge River Basin is located in the northern part of Mongolia. It originates from the northern slopes of the Khan Gai Mountain in Mongolia and finally empties into Lake Baikal in Russia. The Selenge River is the longest and most abundant river in Mongolia, with a total length of 1024 km and a basin size of 447,060 km^2^, which accounts for 82% of the drainage area of Baikal Lake^27^. This region comprises 12 provinces (Fig. 7). The land cover type is mainly grassland and it has a semi-arid climate^37^. The basin is rich in precipitation, has a humid climate, and is a major populated area in Mongolia^38^. Statistics show that by 2020, the livestock in this basin accounted for 58% of the national total. Sheep, goats, cattle, horses, and camels are the main animals raised in the region, resulting in a unique “five livestock” structure. Among these, sheep and goats are essential to the animal husbandry products in the region, accounting for most of the total output value of animal husbandry in the region. Cattle and horses are mainly used for farming and transportation, while the number of camels is relatively small.

### Data acquisition and preprocessing

#### Environmental factor extraction

Livestock distribution is influenced by spatial heterogeneity of social environment and resources. Combining the existing research base and the basic national conditions of the Selenge River Basin in Mongolia, we selected eight influential factors to describe the spatial distribution of livestock density based on the principle of quantifiability. The selected environmental factors are shown in Table 3. Population density of pastoral areas was extracted based on MCD12Q1 land cover data, NPP-VIIR night light data and WorldPop data. The areas where livestock are distributed generally meet two conditions: areas near grasslands with population distribution and non-urban areas^39^. Firstly, based on the above two conditions, we extracted the grid of “population density > 0” from WorldPop to represent the population distribution area. We then used an aggregation tool to generate the area proportion data of grassland in 1 km pixel from the reclassified MCD12Q1 land cover data, and retain an area with the area proportion of grassland greater than 0; a grid with a night light value of 0 was extracted from the NPP-VIIRS night light data to represent the livestock activity area, and then the population density of pastoral area was further extracted by combining these three indicators. Elevation data were obtained from the shuttle radar topographic mission (SRTM). Slope and aspect were obtained using the Slope and Aspect analysis tool in ArcGIS. Normalized vegetation index (NDVI), surface temperature data (LST), and net primary productivity (NPP) were derived from the Moderate-resolution Imaging Spectroradiometer (MODIS) sensor. In this case, the annual NDVI was calculated by synthesizing the maximum value of MOD13Q1; land surface temperature was calculated by unit conversion ($LST=DN\times 0.02-273.15$) using MOD11A2-LST; based on the obtained NPP layer information, after a series of operations such as filling removal, assigning NoData to 0, and multiplying by the scale factor 0.0001, the unit was converted to gC/m^2^/year to obtain the NPP. The precipitation data were obtained from the Global precipitation measurements (GPM) data. The settlement datas were from OpenStreetMap, and the kernel density tool in ArcGIS spatial analysis was used to generate the settlement density raster data. To improve the efficiency of data acquisition, we used the GEE (Google Earth Engine) platform to download MODIS series data, which were transformed by code and unified in spatial resolution, while other data needed to be projected as raster data and resampled in ArcGIS. Subsequently, all data were projected onto WGS_1984 UTM_zone_48N with a unified spatial resolution of 1 km, and cropped to obtain the data for the Selenge River Basin. Finally, various factors were masked to obtain suitable areas for livestock distribution based on the global suitability distribution map of pastoral areas.

**Table 3:** Datasets used in the study.

Dataset	Variables	Resolution/type	Source
WorldPop (2020)	Population density	Grid, 1000 m spatial resolution	WorldPop (https://hub.worldpop.org/)
MCD12Q1 (2020)	Grassland	Grid, 500 m spatial resolution	LP DACC (https://lpdaac.usgs.gov/products/mcd12q1v006/)
NPP-VIIR Night light (2020)	Light value	Grid, 500 m spatial resolution	Earth Observation Group (https://eogdata.mines.edu/products/vnl/)
SRTMDEM (2020)	Slope/Aspect	Grid, 30 m spatial resolution	United States Geological Survey (USGS) (https://earthexplorer.usgs.gov/)
MOD13A2 (2020)	NDVI	Grid, 1000 m spatial resolution	LP DACC (https://lpdaac.usgs.gov/products/mod13a2v006/)
Precipitation Measurement Mission (GPM) (2020)	Annual precipitation	Grid, 0.1°spatial resolution	GES DISC (https://disc.gsfc.nasa.gov/datasets?keywords=GPM&page=1)
MOD11A2(2020)	Average annual land surface temperature	Grid, 1000 m spatial resolution	LAADS DAAC (https://ladsweb.modaps.eosdis.nasa.gov/)
Settlements	Settlement density	Point features	OpenStreetMap (http://www.openstreetmap.org/)
MOD17A3H (2020)	NPP	Grid, 500 m spatial resolution	LP DACC (https://lpdaac.usgs.gov/products/mod17a3hv006/)
Stock data of sheep & goats	Sheep & goats density	Table	Mongolian Statistical Office (https://www.1212.mn/)
Stock data of cattle	Cattle density		
Stock data of horse	Horse density		
Pasture suitability	–	Grid, 1000 m spatial resolution	United Nations Food and Agriculture Organization (https://data.apps.fao.org/map/catalog)

Since the distribution of livestock is highly correlated with the distribution of population density^40^, using the population density of pastoral areas as an environmental factor helps improve the accuracy of spatial simulation^39^. Slope and aspect were chosen because the natural environment drives the distribution of livestock mainly through the topography, and livestock tend to be distributed in plains and lower terrain areas^13^. To some extent, NPP and NDVI reflect the availability of feed biomass, which has geospatial heterogeneity and is also an important indicator of the geographical spatial distribution of livestock^21^. Precipitation and land surface temperature can reflect climatic differences in different regions. These environmental variables directly affect vegetation growth and indirectly influence livestock distribution by determining the availability of fodder and water in a given area^41,42^. Settlement density also reflects the aggregation of livestock density. The factors we chose are not comprehensive, but are representative and reflect the geographic characteristics of livestock spatial distribution from different perspectives. Therefore, we used natural geographic data and livestock statistics as the basic data and extracted the mask data of each environmental factor based on the global pasture suitability distribution map for subsequent processing and analysis. Before proceeding with the modeling, we conducted a collinearity test using SPSS software, and although RF was shown to be able to handle covariance among multiple variables, we were still cautious to avoid factors with VIF > 5 entering the model.

#### Livestock statistics

Livestock statistics were acquired from the year-end livestock stock data of the Mongolian Statistical Office for 2020, and cattle, sheep, and horse data from various soums in the Selenge River Basin were obtained after sorting. Khutul, Sukhbaatar, Chingeltei, and Bayangol did not contain livestock and were thus excluded from the analysis. We classified to reveal the distribution of general livestock density in the Selenge River Basin, but the number of camel is extremely low, so a spatial model was constructed for four types, namely, total livestock (including sheep, goats, horses, cattle and camels), sheep & goats, horses, and cattle, and spatial distribution maps were obtained. Among them, the total livestock was categorized into various livestock types according to experience, one goat and sheep are all one sheep unit, while cattle and horses were categorized as large livestock, and they are uniformly converted into sheep units according to a large livestock equal to five sheep units for statistics; sheep & goats is simulated according to the statistics of goats and sheep; the simulation data of horse and cattle are not converted into units.

### Random forest (RF)

RF is an ensemble learning method for classification and regression proposed by Leo Breiman in “Machine Learning”^43^ and is an improvement of the decision trees algorithm. Compared with other traditional regression prediction models, RF not only has high prediction accuracy, but can also avoid overfitting^44^, can solve the problem of the number of variables being greater than the number of observations, effectively process high-dimensional data^45,46^, and can evaluate the importance of each feature while analyzing the data.

**Figure 8:**
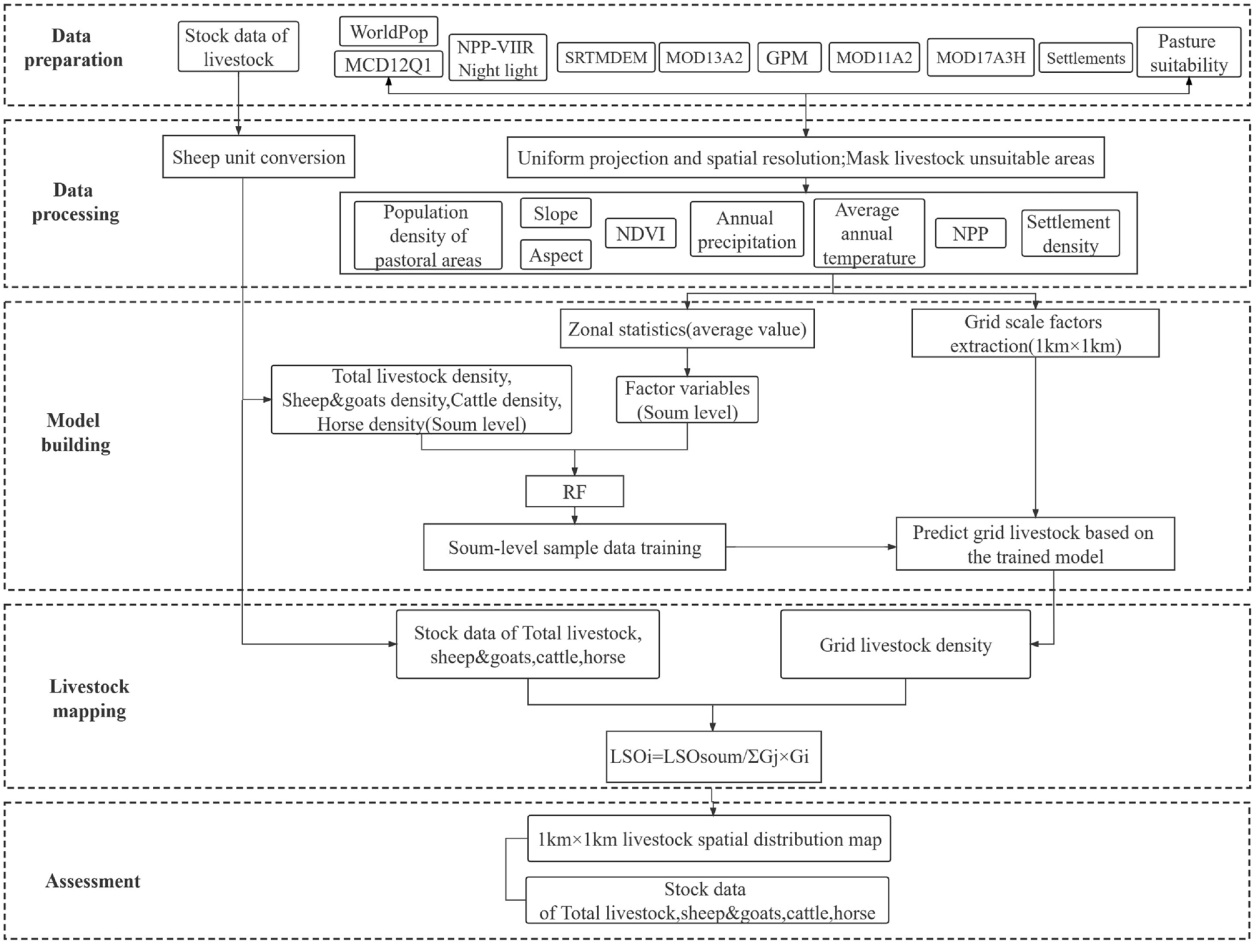
Flowchart of the livestock spatialization process.

In this study, we combined multi-source data and used the RF model to draw the spatial distribution of livestock, which involved five main steps (Fig. 8). (1) Data preparation. Remote sensing data were downloaded and soum-level livestock statistical data were collated, because smaller spatial units (soum) have higher spatial accuracy and better reflect the more informative spatial variation of each livestock class^9^. (2) Data preprocessing. Remote sensing data were preprocessed, including unified coordinate system, spatial resolution, and masking, was performed to identify areas suitable for livestock activity. (3) Model training and prediction. (4) Dasymetric mapping. (5) Accuracy validation of livestock data for each soum. Combined with the existing research data, we believe that the statistical relationship between livestock density and these environmental variables at the soum scale are robust enough to achieve spatial reproduction of livestock statistical data. Therefore, we applied the trained model to the grid scale for prediction and obtained the livestock density data with a spatial resolution of 1 km. To maintain a good agreement between the simulated livestock density and the statistical data, we further used statistical data as the control. Finally, the livestock density gridded data were compared with livestock density statistics at the soum scale to verify the accuracy of the livestock spatialization.

#### Feature data normalization

To eliminate the possible errors caused by unit differences and size differences between multidimensional feature values, the MinMaxScaler() function in the Sklearn software package was used to normalize each factor value, and the data were scaled to the interval^43^. The mathematical prototype of the function is expressed as follows:

1 $${X}_{Mms}=\frac{X-{X}_{Min}}{\left(X-{X}_{Max}\right)-\left(X-{X}_{Min}\right)},$$

where $X$ is the data value, ${X}_{Min}$ is the minimum value of the data, ${X}_{Max}$ is the maximum value of the data, and ${X}_{Mms}$ is the normalized characteristic value. This approach enables the maintenance of the original data distribution state.

#### Model building and training

The Python language and Scikit-learn library were used as programming bases to construct the RF model, combined with the previously processed raster data to obtain the data samples of each soum. The average value of each soum environmental factor was extracted as an independent variable to construct a model, with a spatial resolution of 1 km. For the model dependent variable, livestock density was calculated based on the administrative area and livestock statistical data, and then logarithmically transformed to $LN\left(n+1\right)$ to normalize the distribution of the dependent variable. Using the Scikit-learn library and RandomForestRegressor class, stochastic forest regression models were constructed. According to the 7:3 random allocation principle, the data sample is divided into training set and test set, that is, 70% of the data is selected for training the model and 30% of the sample data is selected as the test set. Using fixed random numbers for partitioning can ensure the randomness of dataset partitioning and the repeatability of experiments, optimize and adjust the model parameters, ensure optimal model performance, and ensure the accuracy of the training results. In this study, we used RandomizedSearchCV to determine the optimal parameters^25^. Different samples have different optimal parameter values, and the optimal values of parameters used to construct the spatial model of livestock in the RF are shown in Table 4.

**Table 4:** Selection of optimal parameters in RF.

Parameter value	Total livestock	Sheep & goats	Cattle	Horse
bootstrap	True	True	False	False
n_estimators	377	544	266	266
min_samples_split	5	2	2	2
min_samples_leaf	2	4	2	2
max_features	sqrt	Auto	sqrt	sqrt
max_depth	7	6	7	7
Accuracy	0.9288	0.9135	0.8926	0.8886

#### Feature importance analysis

RF can evaluate the various features involved in model training, and determine the importance of the features. There are two main methods for evaluating the importance of RF features, the GINI index and out-of-pocket data (OOB) error rate^25^. Compared to the GINI index, the OOB has a wider range of practical applications and yields more accurate prediction results. In the specific implementation idea based on the OOB method, a trained decision tree ${n}_{1}$ is constructed using OOB samples, and the difference between the original value of the sample and the predicted value is used to obtain the out-of-pocket data error ${e}_{1}$, after which the other columns are kept unchanged, permutating or adding noise to the characteristic value of the column $i$ feature to obtain the out-of-pocket data error ${e}_{i2}$. The importance of feature X was calculated using the equation $\sum ({e}_{i2}-{e}_{1})/T$. The greater the change in ${e}_{i2}$ after changing the feature value, the greater the influence of the feature and the more important the feature value. Using OOB to permute or add noise to each feature, an iterative process was carried out to evaluate the $\sum ({e}_{i2}-{e}_{1})/T$ after changing each feature, after which the feature with the higher value was prioritized. This study quantified the importance of each feature in the spatialization of livestock through OOB scoring, which not only reflects the importance of each feature in all features but also the influence of each factor on the spatial distribution of livestock to a certain extent.

#### Livestock gridded density simulation

Livestock gridded density simulation based on a pre-trained livestock spatialization model of the basin. After the masking, eight types of raster data including population density of pastoral areas, slope, aspect, annual precipitation, average annual land surface temperature, NDVI, NPP, and settlement density, were extracted to the grid scale to build the RF feature database. The trained model was then applied to the 1 km grid scale to simulate the density of total livestock, sheep & goats, cattle, and horses, and a spatial distribution map of livestock density with a spatial resolution of 1 km was obtained.

### Dasymetric mapping

Livestock spatialization is the process of transforming livestock density from soum scale to grid scale. In the simulation, we assumed that the influence of environmental factors on livestock density at the two scales was the same, however, notable differences were observed in the spatial distribution characteristics of environmental factor values at the two scales, and errors were found when the model at the soum scale was applied to the grid scale. Since the livestock spatial model is constructed based on the average environmental factors and livestock density at the soum scale, it is necessary to use the statistical data of livestock in each soum to control and redistribute soum-scale livestock at the grid scale^19,20^. The objective was to exponentiate the gridded values of livestock predicted by the model, initially obtain the gridded livestock density prediction data, and then redistribute the grid values according to the total number of livestock in each soum, taking the indexed gridded value data as the weight layer and redistributing the livestock density according to formula (2). The redistributed gridded livestock density was the result of the spatialization at 1 km of livestock density in the Selenge River Basin.

2 $${LSO}_{i}=\frac{{LSO}_{soum}}{{\sum }_{j=1}^{soum}{G}_{i}}\times {G}_{i},$$

where ${LSO}_{i}$ represents the adjusted livestock density value of the grid $i$; ${G}_{i}$ represents the gridded value after the original predicted value is indexed. ${\sum }_{j=1}^{soum}{G}_{i}$ represents the sum of the exponential predicted values of all the grids in the soum where the grid is located. ${LSO}_{soum}$ represents the total of livestock statistical data for soum.

### Accuracy verification

To further verify the accuracy of the simulation results, the simulated values of livestock density of all grids in each soum were counted in different regions, and the errors between the simulated data and the statistical data were calculated. In this study, root mean square error ($RMSE$), absolute error ($MAE$), and coefficient of determination (${R}^{2}$) were used to evaluate the accuracy of the simulation data.

3 $$RMSE=\sqrt{\frac{1}{n}{\sum }_{i=1}^{n}{({P}_{i}-{R}_{i})}^{2}},$$

4 $$MAE=\frac{1}{n}{\sum }_{i=1}^{n}\left|{P}_{i}-{R}_{i}\right|,$$

5 $${R}^{2}=1-\frac{{\sum }_{i=1}^{n}{\left({R}_{i}-{P}_{i}\right)}^{2}}{{\sum }_{i=1}^{n}{\left({R}_{i}-\overline{R }\right)}^{2}},$$

where ${R}_{i}$ is the statistical value of livestock density of soum $i$, ${P}_{i}$ is the simulated value of livestock density of soum $i$, $\overline{R }$ is the statistical average of livestock density of $n$ soum, and $n$ is the total number of samples. Among them, $RMSE$ describes the degree of deviation between simulated livestock values and real statistical values, $MAE$ describes the absolute value of simulated livestock errors, and ${R}^{2}$ measures the degree of fitting between simulated livestock values and real statistical values. The smaller the $RMSE$ and $MAE$, the better the prediction result. The closer ${R}^{2}$ is to 1, the closer the simulated value of the model used is to the true value, and the higher the simulation accuracy.

### Geodetector model

Geodetector is a spatial analysis method based on the principles of geography and statistics, which can detect spatial heterogeneity and reveal the driving force behind a phenomenon by assessing the influence of related factors^47^. This model can analyze the interaction of a single factor and multiple factors in the same spatial scale, which makes the results more scientific. Geodetector includes four parts: interaction detector, factor detector, ecological detector and risk detector^48^. We used factor detector and interaction detector to analyze the impacts of population density in pastoral areas, settlement density, slope, aspect, precipitation, land surface temperature, NPP and NDVI on simulated livestock density.

The working principle of geodetector is that if an independent variable has a significant influence on a dependent variable, then the spatial distribution of the independent variable and the dependent variable should be similar^48^. Among them, the interactive detector in the geographic detector examines whether two factors, when combined, enhance or weaken their effect on livestock density compared to their individual effect^49^. The factor detector in the geodetector is mainly used to analyze the determining force of the independent variable on the dependent variable, and we used the factor detector to quantitatively detect the influence mechanism of each environmental factor on the spatial stratified heterogeneity of sheep & goats, horse and cattle density, which can be measured using the q-statistic^50^:

6 $$q=1-\frac{1}{N{\sigma }^{2}}\sum_{h=1}^{L}{N}_{h}{{\sigma }_{h}}^{2},$$

where $q$ is the explanatory or determining power of each environmental factor on livestock density, and the range of values is 0–1. The larger the $q$- value, the greater the influence of the factor on livestock density and the stronger the explanatory power; $N$ is the number of samples in the study area, and ${N}_{h}$ is the number of samples in strata $h$; ${{\sigma }_{h}}^{2}$ and ${\sigma }^{2}$ are the variance of livestock density in strata $h$ and the study region, respectively; and $L$ is the number of strata. We have spatially discretized the factors before they enter the geodetector analysis.

## Data Availability

The data that supports the findings of this study are available from the corresponding authors upon request.
